# Low serum lipase levels in mothers of children with stunted growth indicate the possibility of low calcium absorption during pregnancy: A cross-sectional study in North Sumatra, Indonesia

**DOI:** 10.1371/journal.pone.0298253

**Published:** 2024-06-06

**Authors:** Dina Keumala Sari, Rina Amelia, Dewi Masyithah, Kraichat Tantrakarnapa

**Affiliations:** 1 Department of Nutrition, Faculty of Medicine, Universitas Sumatera Utara, Medan, North Sumatra, Indonesia; 2 Department of Public Health, Faculty of Medicine, Universitas Sumatera Utara, Medan, North Sumatra, Indonesia; 3 Department of Parasitology, Faculty of Medicine, Universitas Sumatera Utara, Medan, North Sumatra, Indonesia; 4 Department of Social and Environmental Medicine, Faculty of Tropical Medicine, Mahidol University, Bangkok, Thailand; Siddhi Memorial Hospital, NEPAL

## Abstract

Stunting is caused by various factors, including low nutritional intake in the first two years of life. This study aimed to investigate the differences in sociodemographic factors and mineral, vitamin, and enzyme parameters in mothers associated with the occurrence of stunting in children. We conducted a cross-sectional study from September to November 2020 on North Sumatra Island, Indonesia. The data collected included sociodemographic characteristics, pregnancy history, birth history, food intake, and laboratory examinations, including measurements of calcium, iron, zinc, vitamin D, pancreatic amylase, and serum lipase levels. This study included 50 healthy mothers aged 18–50 years old with children aged 2 to 60 months. There was a significant difference in serum calcium levels between the groups of mothers of children with normal and stunted growth (*p* = 0.03, mean difference±standard error (SE) = 0.23±0.12, 95% CI: 0.19–0.45). All of the study subjects were categorized as vitamin D deficient. The mean lipase level in the group of mothers of children with stunted growth was significantly lower than that in the group of mothers of children with normal growth (*p* = 0.02, mean difference±SE = 4.34±1.83, 95% CI: 0.62–8.06). The conclusion was that serum lipase levels were significantly lower in mothers of children with stunted growth compared to mothers of children with normal growth. Serum lipase levels this low are likely to indicate that a mother is unable to meet her child’s calcium needs during pregnancy, increasing the child’s risk of stunted growth.

## Introduction

The World Health Organization (WHO) defines stunting as the failure of growth and development in children due to insufficient nutritional intake over a long period, recurrent infectious diseases, and inadequate psychosocial stimulation [1–7]. Stunting still occurs in developing countries, such as India, Ethiopia, and Indonesia, and this is an important concern because it affects the health of individuals in adulthood and impacts the economy of a country [2–4, 8, 9].

Indonesia still has a high number of individuals with nutritional deficiencies in several provinces, including North Sumatra [10–13]. Numerous diseases due to nutritional disorders such as stunting, hydrocephalus, and various others still occur in this region. The stunting rate for 0- to 60-month-old children in North Sumatra in 2019 was 30.1%, down 2.3% percent from that in 2018 [13, 14]. Children with stunted growth are susceptible to disease and have below-average levels of intelligence and low productivity [15–18].

Stunting is caused by various factors, including low nutritional intake in the first two years of life [10]. A mother’s nutritional status is very important in determining whether a child’s growth is stunted or not [11, 19–21]. The nutritional status of the mother, including the fulfilment of macronutrient and micronutrient needs, will affect the nutritional status of the child, which determines the likelihood of stunting [22–24]. Despite the implementation of programs designed to tackle stunting, cases of stunting are still common. Naturally, this creates the desire to understand the main causes of stunted growth.

The nutritional deficiencies mainly associated with stunted bone growth are calcium and vitamin D deficiencies [25–28]. However, deficiencies of micronutrients, such as minerals and vitamins, are often not detected because patients initially present with subclinical symptoms but will have long-term defects [29–31]. Micronutrient deficiencies are critical, causing disturbances in nutritional status, especially for children, that will last until adulthood [7, 32].

The metabolism of calcium and vitamin D is closely related to their absorption in the body and involves the body’s digestive enzymes [33, 34]. Digestive enzymes support the absorption of fat, and sufficient levels of fat support the absorption of calcium and vitamin D, especially in pregnant mothers; this is important because it affects the nutritional status of children [35]. Based on previous research, further study is needed to explore the role of vitamins, minerals, and enzymes in preventing stunting. Research on enzymes that influence nutrient absorption, including pancreatic amylase and lipase, which are involved in carbohydrate absorption and fat absorption, respectively, has not been widely reported. Research on lipase enzymes that influence the absorption of calcium and vitamin D has also not been reported; for this reason, the serum lipase level was one of the variables included in this study.

The existence of various diseases affects the conditions for providing nutritional intake in childhood. Indonesia, which has been affected by the COVID-19 pandemic since February 2020, has also experienced various economic problems that affect the availability of food [36, 37]. Most people rely on food items that are obtained from the area around their residence [38]. Influential micronutrients from the surrounding soil include minerals, which are from metal and inorganic compounds [39–41].

In this study, we evaluated the factors influencing the occurrence of stunting in children, especially during the COVID-19 pandemic. The factors examined included sociodemographic characteristics, pregnancy history, birth history, nutritional intake, and laboratory tests, including measurements of calcium, iron, zinc, vitamin D, pancreatic amylase, and serum lipase levels. This study focused on mothers with children aged 2 to 60 months old. The results of this study may provide new insights regarding the occurrence of stunting so that prevention measures can be implemented. However, in the initial stages of research, the focus was on vitamins, minerals and enzymes that influence a mother’s nutritional needs.

## Materials and methods

### Study design

This was a cross-sectional study that included 50 mothers and 50 children. We recorded data on sociodemographic characteristics, maternal pregnancy and birth history, anthropometric characteristics of the mothers and children, daily food intake of the mothers and children, and maternal levels of calcium, iron, zinc, vitamin D, pancreatic amylase, and serum lipase. This study also assessed the soil and water content to examine the condition of food sources from the local area.

The determination of the sample size was based on the research hypothesis, an unpaired two-sided hypothesis with comparison testing of two unpaired groups. This study compared the serum vitamin/mineral and enzyme levels between two groups. To determine the sample size, the number of samples taken with a type I error was set at 5%, and a two-sided hypothesis was used. The Zα value was 1.96. The type II error was set at 20%, with Zβ = 0.84. This study used a significance value of 0.005, and the calculation was carried out using the existing hypothesis and by taking the largest sample size.

This research was conducted from September to November 2020 during the COVID-19 pandemic, but all studies were conducted by implementing the relevant health protocols. Health protocols included cleaning tools with a disinfectant after each use, using personal protective equipment, and maintaining a distance of 1–2 metres.

We studied stunting in North Sumatra because of its known high stunting rate. The districts with high rates of stunting include Medan City, Deli Serdang, Langkat, Simalungun, Dairi, Pakpak Barat, Tapanuli Tengah, Mandailing Natal, Padang Lawas, Padang Lawas Utara, Nias, South Nias, West Nias, North Nias, and Gunung Sitoli. This research was conducted in one location at the Community Health Centre for Simpang Dolok Village, Datuk Lima Puluh District, Batu Bara District, Simalungun and Batubara Border, North Sumatra, Indonesia. This research took place in one district to avoid having heterogeneous data.

This study divided all subjects into two groups based on anthropometric examinations of the children (mothers were grouped according to the nutritional status of their child). The examination was carried out by measuring the height for age and determining the nutritional status of the child based on the WHO growth curve. All study subjects were divided into two groups: a group of children with normal growth and a group of children with stunted growth.

### Participants

The research participants were healthy women aged 18–50 years old with children aged 2 to 60 months. The exclusion criteria were as follows: pregnant women; nursing mothers; women with impaired kidney and liver function, chronic disease or other metabolic disorders; and children with chronic diseases. Confounding factors in this study were maternal gastrointestinal disorders and maternal metabolic disorders, which we controlled for in the inclusion and exclusion criteria. Participants were recruited through appeals from the integrated service centre for mothers and children and the community health centre in the target village at the appointed time, and then research was carried out by recording the number of mothers and children who volunteer to participate. The arrival of mothers and children with stunted or normal growth was not determined, but they were randomly assigned at the time of the study.

At the time of participant selection, 62 research subjects were enrolled, but after applying the exclusion criteria, 50 research subjects were included. All subjects were divided into two groups: a group of children with normal growth (32 mothers and their children) and a group of children with stunted growth (18 mothers and their children).

### Instrument

A questionnaire was utilized for the collection of data for this research to determine the characteristics of the research subjects, including age, occupation type, occupation type of their partner/husband, pregnancy history, birth history, and other information. Data regarding the soil and water content were checked in a laboratory.

Soil and water laboratory analyses were carried out to determine soil fertility, which may affect crops in the area. The quality of vegetables and fruits grown in local soil can also be affected. On soil examination, the magnesium level was 87.2 mg/100 g (normal limit: 25–44 mg/100 g), the potassium level was 66.96 mg/100 g (normal limit: 30–250 mg/100 g), the phosphorus level was 0.251 mg/kg (normal limit: 0.3–0.5 mg/100 kg), and the nitrogen content was 0.1% (normal level: 0.06–0.17%). Magnesium, potassium, and nitrogen levels were within normal limits, and only phosphorus levels were lower than normal [42].

Groundwater examination was carried out, and the pH of the water was 4.49 (normal pH is 6.69–7.13); this level indicated that the water was acidic. The ammonium level in the water was 17.48 mg/L (normal level is 1–5 mg/L. The water did not contain faecal coliform bacteria. Based on this examination, the water in this area was of low quality [43, 44].

### Anthropometric examination

Maternal anthropometric measurements included height (TB) and body weight (BW) with standard procedures. Each measurement was made two times, and the average of the measurements was taken. The results of these measurements were used to determine body mass index (BMI). Height was measured by placing a microtoise on the wall at two metres from a flat floor. The subject stood upright in the middle of the microtoise without wearing footwear or socks. The subject faced straight ahead, with their shoulders, buttocks and heels were pressed against the wall and their arms free at their sides. The moving part of the microtoise was carefully lowered so that it touched the top of the head, and the hair was pressed. Height measurements were taken twice; the results were read, averaged and recorded on the form [45]. These measurements were taken alternately following health protocols, meaning that the area was cleaned with disinfectant after each subject was measured.

BW was measured by placing a digital scale on a flat and hard floor without a base. Before weighing, the scale was zeroed. Subjects stood upright in the middle of the scale footrest without wearing footwear or socks and wearing minimal clothing. Weight measurements were carried out twice, and the results were averaged [46]. BMI was calculated by dividing the BW in kilograms by the TB squared in metres (kg/m^2^). The BMI categories for women were as follows: underweight (<18.5 kg/m^2^), normal weight (18.5–22.9 kg/m^2^), overweight (23–24.9 kg/m^2^), obese I (25–29.0 kg/m^2^), and obese II (>30 kg/m^2^) [47].

### Anthropometric measurements of children

Anthropometric measurements of BW in children who were able to stand were performed using digital scales, while digital baby scales were used for children who were unable to stand. Height was determined using a microtoise or longboard. Nutritional status was determined based on the WHO classification of malnutrition in children as underweight, stunting and malnutrition or wasting based on BW and TB measurements for children aged 0–23 months and height for children aged 24–60 months [48]. Nutritional status determination was based on the WHO’s growth curve, with the underweight category (weight for age) comprising underweight and severe underweight. The category was used if the weight for age is less than 2 standard deviations (SDs) below the mean [48].

The stunting category (height for age) comprised stunting and severe stunting. The category was used if the height for age was less than 2 SDs below the mean. The wasting category (weight for height) comprised wasting and severe wasting, and the category was used if the weight for height was less than 2 SDs below the mean [48].

### Laboratory measurement

Laboratory tests were carried out while following the COVID-19 protocols, with the use of complete personal protective equipment. Laboratory tests were performed by taking maternal blood samples to measure calcium, iron, zinc, vitamin D, pancreatic amylase, and lipase levels. Examination of serum calcium in this study was performed using the Arsenazo III method with ARCHITECTc8000 (Abbott, Illinois, United States), and the reagent material was CALCIUM1500T (Abbott). We used a serum sample with stability at 20–25°C. The results of the serum calcium examination were considered either normal (8.4–10.2 mg/dL) or deficient (<8.4 mg/dL).

The examination of serum zinc levels in this study was performed using inductively coupled plasma-mass spectrophotometry (ICP‒MS) with an Agilent 7700 Series ICP‒MS (Agilent Technologies, United States). Serum samples with stability at 2–8°C were used. The results of the serum zinc examination were considered normal at 60–130 mcg/dL or deficient at <60 mcg/dL.

We used ICP‒MS to measure serum iron levels in this study using the Agilent 7700 Series ICP‒MS. A serum iron level of 35–145 mcg/dL was considered normal, while an iron level <35 mcg/dL was considered to indicate deficiency.

Serum amylase levels were measured using spectrometry with an ARCHITECTc8000 (Abbott, Illinois, United States), and an alpha amylase pancreatic reagent was used (Abbott). Serum samples with a stability of 20–25°C were used. The results of the examination of pancreatic serum amylase were considered normal at levels of 13–53 U/L, and deficient at levels < 13 U/L.

Serum lipase levels were measured using spectrophotometry with an ARCHITECTc8000, and the Lipase 778T reagent (Abbott) was used. We used serum with a stability of 20–25°C. Serum lipase levels were considered normal at 13–51 U/L, while deficiency was considered at a level < 13 U/L.

For the examination of vitamin D, namely, serum 25(OH)D levels, the chemiluminescence immune assay (CLIA) method with total Liaison^®^25-OH Vitamin D (Diasorin, United States) was used. We used serum with a stability of 2–8°C. The results of the examination of vitamin D levels were considered as follows: <10 ng/mL indicated deficiency, 10–29 ng/ml indicated insufficiency, and 30–100 ng/mL indicated sufficiency.

### Food recall

Our assessment of nutrient intake was based on food recall for two days (one weekday and one weekend day) and included energy, protein, fat, and carbohydrate intake. Calculations were made using the Nutrisurvey 2007 application, which included Indonesian foods [49]. In the 24-hour recall method, subjects and their parents were asked to recall the subject’s exact food intake by the nutritionist, who was trained in interviewing techniques. Any two-day assessment interview protocol must be standardized and pretested prior to use. Pretesting was undertaken in an area near the study site, using subjects similar to those who would participate in this study. The nutritionist was retrained to minimize interviewer bias.

Nutrisurvey is a nutrition survey and calculations software application originally developed in Germany with an English translation. This application is useful for nutritional analysis and calculating energy needs, diet planning, diet history, meal frequency, identifying nutrients in food, and other functions in carrying out nutritional management therapy. This free application can be used to analyse a person’s food intake and has been validated.

### Statistical analysis

Data were analysed using the IBM-SPSS v11.5 statistical program (IBM Corp., Chicago, IL, United States). Categorical variables are expressed as percentages. Normally distributed continuous variables are expressed as the mean ± SD, and nonnormally distributed continuous variables are expressed as the median (minimum—maximum). To compare the two groups, the independent T test was used if the distribution was normal, and the Mann—Whitney U test was used for data that were nonnormally distributed. The variables are expressed as *p* values, mean differences±standard errors, and 95% confidence intervals (CIs). All data were stored and properly equipped to prevent data loss. Analysis was carried out after the data were completely collected.

### Ethical consideration

This study was conducted according to the guidelines laid down in the Declaration of Helsinki, and all procedures involving research study participants were approved by the Universitas Sumatera Utara Ethical Committee (No. 423/KEP/USU/2020) and clinicaltrials.gov (NCT04669106) (date of document: November 25^th^, 2020). Written and verbal informed consent was obtained from all subjects. Verbal consent was witnessed and formally recorded in the statement sheet after explanation.

## Results

This study included 50 mothers with a mean age of 31.06 ± 6.29 years and children aged 2 to 60 months. Mothers were divided into two groups, namely, mothers with children who had normal growth and mothers with children who had stunted growth (Table 1). The group of mothers aged 20–30 years old had the highest proportion of children with stunted, while the 30–40-year age group had a higher proportion of mothers with children with normal growth.

**Table 1:** Maternal sociodemographic data.

Variable	Mothers of children with normal growth	Mothers of children with stunted growth	*P*
Age			
Mean ± SD (years)	32.41 ± 5.99	28.6 ± 6.25	0.05^a^
Age category (years):			
18–20	1 (3.1%)	1 (5.6%)	0.34^c^
20–30	12 (37.5%)	11 (61.1%)	
30–40	18 (56.3%)	6 (33.3%)	
40–50	1 (3.1%)	0	
Mother’s occupation:			
Midwife	1 (3.1%)	0	0.46^c^
Teacher	1 (3.1%)	1 (5.6%)	
Housewife	24 (75.0%)	17 (94.4)	
Labour	1 (3.1%)	0	
Government employee	5 (15.6)	0	
Husband’s occupation:			
Labour	2 (6.3%)	3 (16.7%)	0.56^c^
Teacher	2 (6.3%)	0	
Fisherman	10 (31.3%)	5 (27.8%)	
Small traders	17 (53.1%)	10 (55.6%)	
Government employee	1 (3.1%)	0	
Mother’s education level:			
No education	0	1 (5.6%)	0.34^c^
Primary School	13 (40.6%)	8 (44.4%)	
Junior High School	4 (12.5%)	3 (16.7%)	
Senior High School	7 (21.9%)	5 (27.8%)	
Bachelor’s degree	8 (25.0%)	1 (2.0%)	
Family income per month:			
Mean ± SD (USD)	93.19 ±62.17	92.73± 48.49	0.98^b^
Family income category:			
≥RMW (USD 173.69)	27 (84.4%)	16 (88.9%)	1.00^c^
≥RMW (USD 173.69)	5 (15.6%)	2 (11.1%)	
Number of children:			
Mean ± SD (count)	2.56 ± 1.4	2.83 ± 1.5	0.90^b^
Age at first pregnancy:			
Mean ± SD (years)	24.38 ± 5.42	24.17 ± 7.08	0.18^b^
Problems during pregnancy:	0	1 (5.6%)	0.26^c^
Fever	3 (9.4%)	0	
Abdominal discomfort	2 (6.3%)	3 (16.7%)	
Nausea and vomiting	1 (3.1%)	0	
Abdominal cramps	26 (81.3%)	14 (77.8%)	
No problems			
History of miscarriage:			
Yes	3 (9.4%)	1 (5.6%)	1.0^c^
No	29 (90.6%)	17 (94.4%)	
Antenatal care:			
Mean ± SD (times per pregnancy)	6.84 ± 2.05	6.06 ± 1.89	0.19^a^

RMW: Regional minimum wage

^a^ Independent t test

^b^ Mann—Whitney U test

^c^ Fisher’s exact test

*significance: *p* < 0.05

Table 1 shows that there were no significant differences between the groups for any sociodemographic variables. The majority of mothers were housewives (69.5%), although the daily income level showed that a lower percentage earned the stipulated regional minimum wage (mean total income±SD: USD 93.03±57.08) per month. Pregnancy history included the number of children (all subjects: mean = 2.66 ± 1.42 children), age at first pregnancy (all subjects: mean ± SD: 24.3 ± 5.99 years), pregnancy problems (67.8% reported no complaints during pregnancy), history of miscarriage (78% had no history of miscarriage), and history of antenatal care (all subjects: mean ± SD: 6.56 ± 2.0 times), and significant differences were not observed between the two groups.

Table 2 provides data on the anthropometric characteristics and nutritional intake of the mothers. All parameters, including the anthropometric parameters, did not show significant differences between the two groups; however, according to the BMI data, mothers of children with normal growth were more likely to be overweight or obese. Moreover, the mean height of mothers was 154.7 ± 5.62 cm, which is slightly lower than the mean height of 159.8 cm for women in Asia (China). Mothers of children with normal growth were taller than mothers of children with stunted growth, but there was no significant difference in height. There was no significant difference in food intake between the two groups.

**Table 2:** Maternal anthropometric and nutritional data.

Variable	Mothers of children with normal growth	Mothers of children with stunted growth	*p*
Mothers			
Body weight	56.94 ± 11.85	57.06 ± 10.29	0.84^b^
Height	155.41 ± 6.06	153.44 ± 4.60	0.21^a^
Body mass index	32.47 ± 4.16	24.14 ± 3.93	0.58^a^
Mother’s body mass index category:			
Underweight	3 (9.4%)	1 (5.6%)	0.89^b^
Normal	15 (46.9%)	9 (50%)	
Overweight	6 (18.8%)	2 (11.1%)	
Obese 1	6 (18.8%)	5 (27.8%)	
Obese 2	2 (6.3%)	1 (5.6%)	
Mother’s intake per day			
Calorie intake	1606.03 ± 298.8	1582.5 ± 269.5	0.78^a^
Carbohydrate intake	233.29 ± 47.7	220.34 ± 42.23	0.33^a^
Protein intake	61.28 ± 12.69	56.32 ± 14.59	0.24^a^
Fat intake	47.68 ± 18.74	55.61 ± 25.19	0.25^a^

^a^ Independent t test

^b^ Mann—Whitney U test

^c^ Fisher’s exact test

*significance: *p* < 0.05

Table 3 shows the birth weight, which showed no significant difference between the two groups. It was reported that nine children had low birth weight (<2500 g), and five of them were from the group of children with stunted growth. Table 3 also shows the data on history of exclusive breastfeeding. Although there were no significant differences between the two groups, the highest percentage of mothers who exclusively breastfed was in the group who had children with normal growth.

**Table 3:** Children anthropometric and nutritional data.

Variable	Children with normal growth	Children with stunted growth	*p*
Age (months)	28.75 ± 18.4	31.89± 21.7	0.23^a^
Birth weight (g)	3200 ± 660	3100 ± 660	0.59
Exclusive breastfeeding			
Yes	19 (59.4%)	9 (50%)	0.56^c^
No	13 (40.6%)	9 (50%)	
Children growth Category			
Weight for age:			
Severe underweight	2 (6.3%)	2 (11.1%)	0.43^c^
Underweight	1 (3.1%)	2 (11.1%)	
Normal	27 (84.4%)	14 (77.8%)	
Obese	2 (6.3%)	0	
Weight for height			
Severe wasting	4 (12.5%)	0	0.11^c^
Wasting	2 (6.3%)	1 (5.6%)	
Normal	24 (75%)	12 (66.7%)	
Obese	2 (6.3%)	5 (27.8%)	
Child’s intake per day			
Calorie intake	907.12 ± 351.81	791.29 ± 215.82	0.16^b^
Carbohydrate intake	207.38 ± 512.05	101.41 ± 38.19	0.25^b^
Protein intake	34.11 ± 15.19	32.38 ± 8.85	0.61^a^
Fat intake	31.37 ± 14.99	28.93 ± 10.77	0.51^a^

^a^ Independent t test

^b^ Mann—Whitney U test

^c^ Fisher’s exact test

*significance: *p* < 0.05

Table 3 also shows the data concerning the nutritional status of the children. Based on weight for age, the two groups of children did not show a significant difference, although it appeared that in the stunting group, 22.2% of the children were underweight or severely underweight, which was higher than the proportion seen in the normal-growth group (9.4%). Based on the stunting category, the stunting group was divided into children who were very short (33.3%) and had short stature (66.7%), whereas in the normal-growth group, most children were considered normal height, although there were also tall children (15.6%).

Table 4 provides the results of measurements of calcium, iron, zinc, vitamin D, pancreatic amylase and lipase levels. These results showed that there was a significant difference in serum calcium levels between the groups of mothers of children with normal and stunted growth and lower serum calcium levels in the group of mothers of children with normal growth (*p* = 0.03, mean difference±standard error (SE) = 0.23±0.12, 95% CI: 0.02–0.45).

**Table 4:** Differences in the nutritional data.

Variable	Mothers of children with normal growth	Mothers of children with stunted growth	*P*
Calcium serum level (mg/dL)	8.82 ± 0.35	9.05 ± 0.37	0.03*^b^
Calcium category:			
Deficiency	2 (6.3%)	0(0%)	0.53^c^
Normal	30 (93.8%)	18 (100%)	
Serum iron level (mcg/dL)	90.25 ± 44.38	94.72 ± 28.91	0.67^a^
Iron category:			
Low	1 (3.1%)	0(0%)	0.54^c^
Normal	27 (84.4%)	17 (94.4%)	
High	4 (12.5%)	1 (5.6%)	
Serum zinc level (mcg/dL)	71.84 ± 7.97	75.0 ± 9.37	0.24^a^
Zinc category:			
Low	2 (6.3%)	1 (5.6%)	1.0^c^
Normal	30 (93.8%)	17 (94.4%)	
Serum vitamin D level (ng/mL)	19.0 ±4.51	20.1 ± 5.14	0.45^a^
Vitamin D category:			
Deficiency	0 (0%)	1 (5.6%)	0.36^c^
Insufficiency	32 (100%)	17 (94.4%)	
Sufficiency	0 (0%)	0 (0%)	
Amylase pancreatic (U/L)	26.03 ± 5.11	25.44 ± 8.03	0.78^a^
Amylase category:			
Low	0 (0%)	0 (0%)	-
Normal	32 (100%)	18 (100%)	
Lipase (U/L)	20.06 ± 6.61	15.72 ± 6.01	0.03*^a^
Lipase category:			
Low	4 (12.5%)	7 (38.9%)	0.04*^c^
Normal	28 (87.5%)	11 (61.1%)	

^a^ Independent t test

^b^ Mann—Whitney U test

^c^ Fisher’s exact test

*significance: *p* < 0.05

Likewise, with the calcium classification, it was seen that 6.3% of mothers in the group of with children with normal growth had calcium deficiency, but there was no significant difference between groups. The parameters of serum iron and zinc levels did not show significant differences between the two groups.

Apart from calcium, serum vitamin D levels did not show any significant difference between the two groups; however, none of the study subjects were categorized as vitamin D deficient (all subjects: mean±SD: 19.0 ± 4.5 ng/mL). However, the levels of calcium and serum 25(OH)D in the group of mothers who had children with stunted growth were higher than those in the group of mothers who had children with normal growth.

With regard to the enzyme examination, serum pancreatic amylase levels did not show any significant differences, but significant differences were seen in serum lipase levels. The mean lipase level in the group of mothers who had children with stunted growth was significantly lower than that in the group of mothers who had children with normal growth (*p* = 0.02, median = 4.34, 95% CI: 0.62–8.06). There was no significant correlation between calcium and lipase (*p* = 0.51) or between calcium and vitamin D (*p* = 0.067) levels.

## Discussion

We investigated factors associated with stunting in 18 children who exhibited stunted growth in a village North Sumatra, Indonesia. A stunting prevention program has been carried out; however, a reanalysis of why cases of stunting are still occurring, despite there being decreased stunting rates in the surrounding area, is required. There are various causes of stunting, such as maternal nutritional status, anaemia, inadequate weight gain during pregnancy, short pregnancy duration, and a very young age at first pregnancy [1, 50–54]. In addition to these factors, there are other fundamental factors, such as low maternal education levels, poor sanitation, and not being exclusively breastfed [55–58]. In our study, there were no differences in sociodemographic characteristics (education level, family income, etc.) among children with normal growth and stunted growth. For this reason, it is necessary to examine the underlying factors of the incidence of stunting in toddlers.

Our study focused on the nutritional status of mothers who have children under five years old. To examine maternal nutritional status, we measured calcium, iron, and zinc levels, deficiencies of which are associated with growth problems and anaemia. An important vitamin related to metabolism is vitamin D, and its measurement requires an understanding of the interaction between calcium and vitamin D. Moreover, the function of the maternal digestive system is related to metabolism and vitamins, so disturbances that occur can affect mineral and vitamin activity.

Stunting is a disease related to long-term nutritional deficiency, especially during the first 1000 days of a child’s life [59–63]. This nutritional deficiency is caused by a lack of macronutrients and micronutrients [64, 65]. Macronutrient diseases are caused by a deficiency of protein, fat and carbohydrates [63, 64]. Children with these deficiencies usually show clinical symptoms that directly affect their nutritional status [64, 66–68]. However, micronutrient deficiencies of minerals and vitamins are often not detected because patients initially present with subclinical symptoms and have long-term defects [69–72]. These defects lead to impaired nutritional status in children and can have serious implications when in adulthood [63].

If babies with stunted growth can catch up and grow quickly, the stunting rate can be reduced and the stunting cycle can be broken between generations. Although most babies with stunting at an early age catch up in growth after the age of two years and during adolescence, there are some metabolic disorders that can occur in adulthood [63, 73, 74]. As a result of the pursuit for growth in height after a delay in early growth, the growth of other organs may be stunted, and cognitive development is also affected, which is linked to obesity in adulthood and associated with diabetes and heart disease [60–63, 75].

Deficiencies in calcium and vitamin D are related to bone growth inhibition, but the metabolism of calcium and vitamin D is closely related to absorption in the body, including the role of the body’s digestive enzymes [33, 76–78]. Our study found that there was a significant difference in serum calcium levels between the two groups; mothers of children with stunted growth had significantly higher calcium levels than mothers of children with normal growth, even though both groups were classified as having calcium levels within the normal range. Serum lipase levels were lower in mothers of children with stunted growth. While our study showed that serum 25(OH)D representing circulating vitamin D were not normal, most cases involved vitamin D insufficiency and deficiency, although no correlation was found between any of these minerals and vitamins, which may be related to the small sample size.

Stunting is a result of long-term nutrient deficiency. All types of nutrient deficiencies can be related. Calcium and vitamin D are two nutrients that affect bone mineral density during pregnancy and a child’s development [70–72, 79, 80]. Maternal nutritional status greatly affects the nutritional status of children during the first 1000 days of life, including the availability of maternal calcium, which will affect foetuses’ calcium levels during pregnancy [63, 81, 82]. Our study found that mothers who had children with stunted growth had high serum calcium levels, low serum vitamin D levels, and low serum lipase levels. Mothers of children with normal growth had low serum calcium levels but were still classified as normal, low serum vitamin D levels, and significantly different serum lipase levels.

During pregnancy, the mother provide calcium to the foetus for growth and development [72, 76, 83–85]. During this period, there are major changes in the body to meet calcium needs and maintain maternal homeostasis [69, 79, 86, 87]. Calcium homeostasis is a complex process involving calcium and parathyroid hormone, calcitonin, and 1,25-dihydroxyvitamin D3 [70–72]. The total serum calcium concentration decreases during pregnancy, and there is increased demand for haemodilution along with a decrease in albumin levels [72]. However, constant serum calcium levels will be maintained through the homeostasis control mechanism so that the body’s calcium needs can be met [83–85, 88, 89].

Homeostasis affects the absorption of calcium, and during pregnancy, there is an increase in intestinal calcium absorption [76, 83, 90]. Calcium absorption is also influenced by fat [76–78, 90, 91]. Fat intake can stimulate calcium absorption depending on the type and amount of fat [77]. Previous research has suggested that a high-fat diet may aid calcium absorption in human and nonhuman animal studies [78]. A high intake of saturated fat causes hyperpermeability of the intestinal membrane, which increases the passive entry of calcium [76, 77].

Calcium ions play an important role in calcium absorption, affecting the rate of lipid hydrolysis [77]. The digestion of fat from long-chain fatty acids is more difficult if there is no support from lipase as a fat-breaking enzyme [77, 91]. Calcium precipitates the accumulated free fatty acids, thereby releasing them from the surface of the lipid granules and making it easier for the lipases to emulsify the lipids [78, 90, 91]. Calcium ions can increase the rate of lipolysis through this mechanism [76]. The deposits formed between calcium and long-chain saturated fatty acids are more difficult to absorb, thus decreasing lipid bioavailability [76, 90].

If the levels of lipase produced by the liver are increased in the blood, then the number of osteoblasts decreases, and the number of osteoclasts increases [33, 86]. Osteoclasts are needed for bone resorption in pregnant women, which contributes to meeting foetal calcium needs [86, 92]. This mechanism is also influenced by parathyroid hormone-related peptide (PTHrP), which increases bone resorption in pregnant women [70, 86]. This can be seen in the increase in PTHrP levels in breast milk [69–72, 86, 93]. In our study, serum lipase levels were significantly decreased in the group of mothers of children with stunted growth, which would lead to an increase in the number of osteoblasts and a decrease in the number of osteoclasts. As a result of this decrease in the number of osteoclasts, the mother would have difficulty meeting the foetus’s calcium needs because the calcium in the mother’s bones cannot be degraded; therefore, the foetus would be born with a low calcium state, causing stunting. This mechanism could be proposed to understand the role of lipase in the occurrence of stunting.

In this study, the serum lipase levels of mothers of children with normal growth were higher than those of mothers of children with stunted growth. The possible mechanism is that high lipase levels can help fat absorption and increase the absorption of calcium from food before pregnancy and during pregnancy. If a mother’s serum lipase level is low, then optimal fat absorption will not occur, which will ultimately inhibit calcium absorption. The possible mechanism explaining stunted growth in children is that their mothers had low lipase levels and were unable to meet the foetal needs for calcium due to a decreased number of osteoclasts, even though the serum calcium levels were normal. High lipase levels lead to high fat absorption, which corresponds to the absorption rate of calcium within the foetal body; therefore, in the event of low lipase levels in mothers, obstructed calcium absorption could lead to stunted growth in children. This could be coupled with the suboptimal absorption of calcium due to low serum vitamin D levels.

The nutritional status of a women is important in every stage of her reproductive period, not only prenatally, including not only her body mass index or height but also her vitamin/mineral and enzyme status after giving birth. This can describe the condition of mothers prenatally and during pregnancy. However, the status of vitamins, minerals and enzymes in mothers deserves attention because the possibility of a mother becoming pregnant again is very high. Moreover, mothers may not pay attention to the interval between the birth of one child and another. This condition is one of the reasons why examination of vitamin/mineral and enzyme status is a main focus and is carried out after birth.

Limitations in this study include the small sample size and the lack of significance of our results (mostly); these differences might be considered a result of normal individual variation rather than group differences. We also did not test the mothers when they were pregnant, so their lipase levels might have changed in the period between birth and our study. Another limitation is that there were no data on maternal parathyroid hormone, calcium, and vitamin D levels and lipase levels in children. Our findings can form the basis for further research, although this study was limited by the small sample size, single study location, and the lack of analysis of the surrounding soil and climate factors. There are still many factors that must be studied to establish the mechanism of calcium regulation in mothers who have children with stunted growth. It is hoped that this research can be generalized to a wider population based on the research methodology used, including the sampling methods and data collection, which were carried out correctly.

## Conclusions

Serum lipase levels were significantly lower in the group of mothers of children with stunted growth when compared with the group of mothers of children with normal growth in one village in North Sumatra, Indonesia. A low serum lipase level probably results in a mother being unable to meet the calcium needs of her child during pregnancy, increasing the child’s risk of stunted growth. Serum calcium levels were significantly higher in the group of mothers of children with stunted growth, possibly due to the interaction among calcium, lipase and vitamin D, which was found to be low in all study subjects. It is hoped that the results of this study will provide further understanding of the role of lipase in calcium metabolism so that programs for the early prevention of growth stunting can be implemented for pregnant women.

## Supporting information

S1 Table Maternal sociodemographic data. (PDF)

S2 Table Maternal anthropometric data. (PDF)

S3 Table Children anthropometric and nutritional data. (PDF)

S4 Table Differences in the nutritional data. (PDF)

S1 Dataset Data set. (SAV)

S1 Data Water examination data. (PDF)

S2 Data Soil examination data. (PDF)

S1 Checklist Strobe check. (PDF)

## References

[1] Bogale B, Gutema BT, Chisha Y. Prevalence of stunting and its associated factors among children of 6–59 months in Arba Minch health and demographic surveillance site (HDSS), Southern Ethiopia: a community-based cross-sectional study. J Environ Public Health. 2020;2020: 9520973. doi: 10.1155/2020/9520973 32280353 PMC7115144

[2] Dhami MV, Ogbo FA, Osuagwu UL, Ugboma Z, Agho KE. Stunting and severe stunting among infants in India: the role of delayed introduction of complementary foods and community and household factors. Glob Health Action. 2019;12: 1638020. doi: 10.1080/16549716.2019.1638020 31333077 PMC7011976

[3] Dong Y, Bennett K, Jan C, Dong B, Zou Z, Hu P, et al. Subnational variation of stunting, wasting and malnutrition in Chinese primary-school children between 2010 and 2014: urban-rural disparity. Public Health Nutr. 2019;22: 2043–2054. doi: 10.1017/S1368980019000235 30827292 PMC10273615

[4] Gebru KF, Haileselassie WM, Temesgen AH, Seid AO, Mulugeta BA. Determinants of stunting among under-five children in Ethiopia: a multilevel mixed-effects analysis of 2016 Ethiopian demographic and health survey data. BMC Pediatr. 2019;19: 176. doi: 10.1186/s12887-019-1545-0 31153381 PMC6544992

[5] Berhe K, Seid O, Gebremariam Y, Berhe A, Etsay N. Risk factors of stunting (chronic undernutrition) of children aged 6 to 24 months in Mekelle city, Tigray region, North Ethiopia: an unmatched case-control study. PLoS One. 2019;14: e0217736. doi: 10.1371/journal.pone.0217736 31181094 PMC6557492

[6] Angdembe MR, Dulal BP, Bhattarai K, Karn S. Trends and predictors of inequality in childhood stunting in Nepal from 1996 to 2016. Int J Equity Health. 2019;18: 42. doi: 10.1186/s12939-019-0944-z 30836975 PMC6402091

[7] Beal T, Le DT, Trinh TH, Burra DD, Huynh T, Duong TT, et al. Child stunting is associated with child, maternal, and environmental factors in Vietnam. Matern Child Nutr. 2019;15: e12826. doi: 10.1111/mcn.12826 30958643 PMC6859968

[8] Emerson E, Savage A, Llewellyn G. Prevalence of underweight, wasting and stunting among young children with a significant cognitive delay in 47 low-income and middle-income countries. J Intellect Disabil Res. 2020;64: 93–102. doi: 10.1111/jir.12698 31845425

[9] Getaneh Z, Melku M, Geta M, Melak T, Hunegnaw MT. Prevalence and determinants of stunting and wasting among public primary school children in Gondar town, Northwest, Ethiopia. BMC Pediatr. 2019;19: 207. 31238889 10.1186/s12887-019-1572-x PMC6591879

[10] Hastuti, Hadju V, Citrakesumasari, Maddeppungeng M. Stunting prevalence and its relationship to birth length of 18–23 months old infants in Indonesia. Enferm Clin. 2020;30: 205–209.

[11] Rachmi CN, Agho KE, Li M, Baur LA. Stunting, underweight and overweight in children aged 2.0–4.9 years in Indonesia: prevalence trends and associated risk factors. PLoS One. 2016;11: e0154756. doi: 10.1371/journal.pone.0154756 27167973 PMC4864317

[12] Ramli CN, Agho KE, Inder KJ, Bowe SJ, Jacobs J, Dibley MJ. Prevalence and risk factors for stunting and severe stunting among under-fives in North Maluku province of Indonesia. BMC Pediatr. 2009;9: 64. doi: 10.1186/1471-2431-9-64 19818167 PMC2765943

[13] Kementerian Kesehatan Republik Indonesia. Hasil utama RISKESDAS 2018. Jakarta: Kemenkes RI; 2018.

[14] Kementerian Kesehatan Republik Indonesia. 1 dari 3 balita Indoneisa derita stunting. 2018 [cited 13 December 2020]. http://www.p2ptm.kemkes.go.id/artikel-sehat/1-dari-3-balita-indonesia-derita-stunting.

[15] Goudet SM, Bogin BA, Madise NJ, Griffiths PL. Nutritional interventions for preventing stunting in children (birth to 59 months) living in urban slums in low- and middle-income countries (LMIC). Cochrane Database Syst Rev. 2019;6: CD011695. doi: 10.1002/14651858.CD011695.pub2 31204795 PMC6572871

[16] Hein AK, Hong SA, Puckpinyo A, Tejativaddhana P. Dietary diversity, social support and stunting among children aged 6–59 months in an internally displaced persons camp in Kayin State, Myanmar. Clin Nutr Res. 2019;8: 307–317. doi: 10.7762/cnr.2019.8.4.307 31720256 PMC6826059

[17] Irenso AA, Dessie Y, Berhane Y, Assefa N, Canavan CR, Fawzi WW. Prevalence and predictors of adolescent linear growth and stunting across the urban-rural gradient in eastern Ethiopia. Trop Med Int Health. 2020;25: 101–110. doi: 10.1111/tmi.13341 31710743

[18] Kuhn-Santos RC, Suano-Souza FI, Puccini RF, Strufaldi MWL. Factors associated with excess weight and stunting in schoolchildren born with low birth weight. Cien Saude Colet. 2019;24: 361–370. 30726369 10.1590/1413-81232018242.30702016

[19] Ban L, Guo S, Scherpbier RW, Wang X, Zhou H, Tata LJ. Child feeding and stunting prevalence in left-behind children: a descriptive analysis of data from a central and Western Chinese population. Int J Public Health. 2017;62: 143–151. doi: 10.1007/s00038-016-0844-6 27318527 PMC5288445

[20] Berhanu G, Mekonnen S, Sisay M. Prevalence of stunting and associated factors among preschool children: a community based comparative cross sectional study in Ethiopia. BMC Nutr. 2018;4: 28. doi: 10.1186/s40795-018-0236-9 32153889 PMC7050938

[21] Berti PR, Leonard WR, Berti WJ. Stunting in an Andean community: prevalence and etiology. Am J Hum Biol. 1998;10: 229–240. doi: 10.1002/(SICI)1520-6300(1998)10:2&lt;229::AID-AJHB8&gt;3.0.CO;2-F 28561441

[22] Trowbridge FL. Prevalence of growth stunting and obesity: pediatric nutrition surveillance system, 1982. MMWR CDC Surveill Summ. 1983;32: 23SS–26SS. 6427593

[23] Wang A, Scherpbier RW, Huang X, Guo S, Yang Y, Josephs-Spaulding J, et al. The dietary diversity and stunting prevalence in minority children under 3 years old: a cross-sectional study in forty-two counties of Western China. Br J Nutr. 2017;118: 840–848. doi: 10.1017/S0007114517002720 29189194

[24] Wessells KR, Brown KH. Estimating the global prevalence of zinc deficiency: results based on zinc availability in national food supplies and the prevalence of stunting. PLoS One. 2012;7: e50568. doi: 10.1371/journal.pone.0050568 23209782 PMC3510072

[25] Gebru TT, Tesfamichael YA, Bitow MT, Assefa NE, Abady GG, Mengesha MB, et al. Stunting and associated factors among under-five children in Wukro town, Tigray region, Ethiopia: a cross sectional study. BMC Res Notes. 2019;12: 504. doi: 10.1186/s13104-019-4535-2 31412922 PMC6693130

[26] Haszard JJ, Diana A, Daniels L, Houghton LA, Gibson RS. Development of a nutrient quality score for the complementary diets of Indonesian infants and relationships with linear growth and stunting: a longitudinal analysis. Br J Nutr. 2019;122: 71–77. doi: 10.1017/S0007114519000813 30975226

[27] Khan S, Zaheer S, Safdar NF. Determinants of stunting, underweight and wasting among children <5 years of age: evidence from 2012–2013 Pakistan demographic and health survey. BMC Public Health. 2019;19: 358. 30935382 10.1186/s12889-019-6688-2 PMC6444880

[28] Kovacs CS. Calcium and phosphate metabolism and related disorders during pregnancy and lactation. In: Feingold KR, Anawalt B, Boyce A, Chrousos G, Dungan K, Grossman A, et al., editors. Endotext. South Dartmouth (MA): MDText.com, Inc.; 2000.

[29] Okazaki R. Body weight and bone/calcium metabolism. Obesity and vitamin D. Clin Calcium. 2018;28: 947–956. 29950548

[30] Sakuma M, Suzuki A, Kikuchi M, Arai H. Soymilk intake has desirable effects on phosphorus and calcium metabolism. J Clin Biochem Nutr. 2018;62: 259–263. doi: 10.3164/jcbn.17-79 29892166 PMC5990409

[31] Shridhar K, Kinra S, Gupta R, Khandelwal S, Prabhakaran D, Cox SE, et al. Serum calcium concentrations, chronic inflammation and glucose metabolism: a cross-sectional analysis in the Andhra Pradesh children and parents study (APCaPS). Curr Dev Nutr. 2019;3: nzy085. doi: 10.1093/cdn/nzy085 30891537 PMC6416530

[32] Fadare O, Mavrotas G, Akerele D, Oyeyemi M. Micronutrient-rich food consumption, intra-household food allocation and child stunting in rural Nigeria. Public Health Nutr. 2019;22: 444–454. doi: 10.1017/S1368980018003075 30501658 PMC10260593

[33] Wang X, Greilberger J, Jurgens G. Calcium and lipoprotein lipase synergistically enhance the binding and uptake of native and oxidized LDL in mouse peritoneal macrophages. Atherosclerosis. 2000;150: 357–363. doi: 10.1016/s0021-9150(99)00413-x 10856527

[34] Soma MR, Gotto AM, Ghiselli G. Rapid modulation of rat adipocyte lipoprotein lipase: effect of calcium, A23187 ionophore, and thrombin. Biochim Biophys Acta. 1989;1003: 307–314. doi: 10.1016/0005-2760(89)90237-3 2545263

[35] Masaka M, Okuda H, Fujii S. Effect of calcium ion on lipase-fat complex formation. J Biochem. 1972;72: 1565–1566. doi: 10.1093/oxfordjournals.jbchem.a130048 4352627

[36] Gugus Tugas Penanganan Covid 19 Indonesia. Data penyebaran covid 19 Indonesia. 2020 [cited 20 May 2020]. https://covid19.go.id.

[37] Lake MA. What we know so far: COVID-19 current clinical knowledge and research. Clin Med (Lond). 2020;20: 124–127. doi: 10.7861/clinmed.2019-coron 32139372 PMC7081812

[38] Meo SA, Al-Khlaiwi T, Usmani AM, Meo AS, Klonoff DC, Hoang TD. Biological and epidemiological trends in the prevalence and mortality due to outbreaks of novel coronavirus COVID-19. J King Saud Univ Sci. 2020;32: 2495–2499. doi: 10.1016/j.jksus.2020.04.004 32292261 PMC7144605

[39] Lu M, Liu Y, Shao M, Tesfaye GC, Yang S. Associations of iron intake, serum iron and serum ferritin with bone mineral density in women: the national health and nutrition examination survey, 2005–2010. Calcif Tissue Int. 2020;106: 232–238. doi: 10.1007/s00223-019-00627-9 31754762

[40] Gupta AP, Bhandari B, Gupta A, Goyal S. Mineral content of breast milk from north Indian mothers giving birth preterm and at term—implication for mineral nutrition of preterm infants. J Trop Pediatr. 1984;30: 286–288. doi: 10.1093/tropej/30.5.286 6512915

[41] Bouain N, Krouk G, Lacombe B, Rouached H. Getting to the root of plant mineral nutrition: combinatorial nutrient stresses reveal emergent properties. Trends Plant Sci. 2019;24: 542–552. doi: 10.1016/j.tplants.2019.03.008 31006547

[42] Fernaández-Sanjurjo MJ, Alvarez-Rodriáguez E, Nuánñez-Delgado A, Fernaández-Marcos ML, Romar-Gasalla A. Nitrogen, phosphorus, potassium, calcium and magnesium release from two compressed fertilizers: column experiments. Solid Earth. 2014;5: 1351–1360.

[43] Zhu F, He S, Liu T. Effect of pH, temperature and co-existing anions on the removal of Cr(VI) in groundwater by green synthesized nZVI/Ni. Ecotoxicol Environ Saf. 2018;163: 544–550. doi: 10.1016/j.ecoenv.2018.07.082 30077151

[44] Song Y, Mao G, Gao G, Bartlam M, Wang Y. Structural and functional changes of groundwater bacterial community during temperature and pH disturbances. Microb Ecol. 2019;78: 428–445. doi: 10.1007/s00248-019-01333-7 30706112

[45] Gibson JC, Temple VA, Anholt JP, Gaul CA. Nutrition needs assessment of young special Olympics participants. J Intellect Dev Disabil. 2011;36: 264–268. doi: 10.3109/13668250.2011.617360 22007752

[46] Kohn JB. Adjusted or ideal body weight for nutrition assessment? J Acad Nutr Diet. 2015;115: 680. doi: 10.1016/j.jand.2015.02.007 25819523

[47] WRPO WHO. The Asia-Pacific perspective: redefining obesity and its intervention. Australia: WRPO WHO; 2000.

[48] World Health Organization. Child growth standard. Geneva: World Health Organization; 2020.

[49] Erhardt J, Gross R. Nutrisurvey and calculations. 2007 [cited 2 April 2010]. http://www.nutrisurvey.de.

[50] Bogale TY, Bala ET, Tadesse M, Asamoah BO. Prevalence and associated factors for stunting among 6–12 years old school age children from rural community of Humbo district, Southern Ethiopia. BMC Public Health. 2018;18: 653. 29793479 10.1186/s12889-018-5561-z PMC5968559

[51] Bose K, Bisai S. Prevalence of underweight and stunting among school children in West Bengal. Indian J Pediatr. 2008;75: 1272. doi: 10.1007/s12098-008-0167-6 18810360

[52] Abate BB, Kassie AM, Kassaw MW, Zemariam AB, Alamaw AW. Prevalence and determinants of stunting among adolescent girls in Ethiopia. J Pediatr Nurs. 2020;52: e1–e6. doi: 10.1016/j.pedn.2020.01.013 32029327

[53] Adedeji IA, Bashir MF, Shwe DD, John C. Prevalence and correlates of stunting among the school-age population in North-Central Nigeria. Pan Afr Med J. 2018;31: 170. doi: 10.11604/pamj.2018.31.170.15763 31086623 PMC6488238

[54] Adenuga WU, Obembe TA, Odebunmi KO, Asuzu MC. Prevalence and determinants of stunting among primary school children in rural and urban communities in Obafemi Owode local government area, Southwestern Nigeria. Ann Ib Postgrad Med. 2017;15: 7–15. 28970765 PMC5598447

[55] Adhikari RP, Shrestha ML, Acharya A, Upadhaya N. Determinants of stunting among children aged 0–59 months in Nepal: findings from Nepal demographic and health survey, 2006, 2011, and 2016. BMC Nutr. 2019;5: 37. 32153950 10.1186/s40795-019-0300-0 PMC7050935

[56] Briend A. The complex relationship between wasting and stunting. Am J Clin Nutr. 2019;110: 271–272. doi: 10.1093/ajcn/nqz050 31172176

[57] Budge S, Parker AH, Hutchings PT, Garbutt C. Environmental enteric dysfunction and child stunting. Nutr Rev. 2019;77: 240–253. doi: 10.1093/nutrit/nuy068 30753710 PMC6394759

[58] Dake SK, Solomon FB, Bobe TM, Tekle HA, Tufa EG. Predictors of stunting among children 6–59 months of age in Sodo Zuria district, South Ethiopia: a community based cross-sectional study. BMC Nutr. 2019;5: 23. 32153936 10.1186/s40795-019-0287-6 PMC7050694

[59] Cordon A, Asturias G, De Vries T, Rohloff P. Advancing child nutrition science in the scaling up nutrition era: a systematic scoping review of stunting research in Guatemala. BMJ Paediatr Open. 2019;3: e000571. doi: 10.1136/bmjpo-2019-000571 32099904 PMC7015046

[60] Reilly JJ, Martin A, Hughes AR. Early-life obesity prevention: critique of intervention trials during the first one thousand days. Curr Obes Rep. 2017;6: 127–133. doi: 10.1007/s13679-017-0255-x 28434107 PMC5487951

[61] Aldana-Parra F, Olaya G, Fewtrell M. Effectiveness of a new approach for exclusive breastfeeding counselling on breastfeeding prevalence, infant growth velocity and postpartum weight loss in overweight or obese women: protocol for a randomized controlled trial. Int Breastfeed J. 2020;15: 2. doi: 10.1186/s13006-019-0249-2 31921328 PMC6945425

[62] Said-Mohamed R, Micklesfield LK, Pettifor JM, Norris SA. Has the prevalence of stunting in South African children changed in 40 years? A systematic review. BMC Public Health. 2015;15: 534. 26044500 10.1186/s12889-015-1844-9 PMC4456716

[63] Agosti M, Tandoi F, Morlacchi L, Bossi A. Nutritional and metabolic programming during the first thousand days of life. Pediatr Med Chir. 2017;39: 157. doi: 10.4081/pmc.2017.157 28673078

[64] Ghosh S. Protein quality in the first thousand days of life. Food Nutr Bull. 2016;37: S14–S21. doi: 10.1177/0379572116629259 27005491

[65] Santos KD, Traebert J, Piovezan AP, Silva JD. Relevance of the first thousand days of life to the development of wheezing in children aged 6–7 years. Allergol Immunopathol (Madr). 2020;48: 270–280. doi: 10.1016/j.aller.2019.12.007 32284262

[66] Mohammed SH, Larijani B, Esmaillzadeh A. Concurrent anemia and stunting in young children: prevalence, dietary and non-dietary associated factors. Nutr J. 2019;18: 10. doi: 10.1186/s12937-019-0436-4 30791904 PMC6385383

[67] Marino LV, Magee A. A cross-sectional audit of the prevalence of stunting in children attending a regional paediatric cardiology service. Cardiol Young. 2016;26: 787–789. doi: 10.1017/S1047951115001778 26361220

[68] Imam A, Hassan-Hanga F, Sallahdeen A, Farouk ZL. A cross-sectional study of prevalence and risk factors for stunting among under-fives attending acute malnutrition treatment programmes in north-western Nigeria: should these programmes be adapted to also manage stunting? Int Health. 2021;13: 262–271. doi: 10.1093/inthealth/ihaa043 32780808 PMC8079315

[69] Grizzo FM, Da Silva Martins J, Pinheiro MM, Jorgetti V, Carvalho MD, Pelloso SM. Pregnancy and lactation-associated osteoporosis: bone histomorphometric analysis and response to treatment with zoledronic acid. Calcif Tissue Int. 2015;97: 421–425. doi: 10.1007/s00223-015-0028-z 26108650

[70] Ajong AB, Kenfack B, Ali IM, Yakum MN, Aljerf L, Telefo PB. Hypocalcaemia and calcium intake in pregnancy: a research protocol for critical analysis of risk factors, maternofoetal outcomes and evaluation of diagnostic methods in a third-category health facility, Cameroon. PLoS One. 2020;15: e0241812. doi: 10.1371/journal.pone.0241812 33152011 PMC7644052

[71] Dai W, Deng X, Li L, Qiu J, Mao B, Shao Y, et al. An observational study on calcium supplementation and dietary intake during pregnancy on low birth weight and small for gestational age. Public Health Nutr. 2021;24: 622–631. 10.1017/S1368980020004425 PMC11574823 33143813

[72] Pentyala S, Rahman A, Mysore P, Tumillo T, Roy A, Pentyala S, et al. Osteoporosis associated with pregnancy. Arch Med. 2015;7: 1–9.

[73] Pentecost M, Ross FC, Macnab A. Beyond the dyad: making developmental origins of health and disease (DOHaD) interventions more inclusive. J Dev Orig Health Dis. 2018;9: 10–14. doi: 10.1017/S2040174417000629 28784190

[74] Tao F. Early life opportunities for promotion of children health. Zhonghua Yu Fang Yi Xue Za Zhi. 2016;50: 105–109. doi: 10.3760/cma.j.issn.0253-9624.2016.02.001 26926715

[75] Francavilla R, Cristofori F, Tripaldi ME, Indrio F. Intervention for dysbiosis in children born by C-section. Ann Nutr Metab. 2018;73: 33–39. doi: 10.1159/000490847 30041184

[76] Hu M, Li Y, Decker EA, McClements DJ. Role of calcium and calcium-binding agents on the lipase digestibility of emulsified lipids using an *in vitro* digestion model. Food Hydrocoll. 2010;24: 7.

[77] Wang Y, Dellatore P, Douard V, Qin L, Watford M, Ferraris RP, et al. High fat diet enriched with saturated, but not monounsaturated fatty acids adversely affects femur, and both diets increase calcium absorption in older female mice. Nutr Res. 2016;36: 742–750. doi: 10.1016/j.nutres.2016.03.002 27262536 PMC4919156

[78] My understanding is that calcium absorption relies on vitamin D and that vitamin D needs fat to be used by the body. If I drink low-fat milk with a low-fat meal, is the milk’s supply of calcium wasted? Mayo Clin Health Lett. 2009;27: 8. 19186307

[79] Wilson RL, Phillips JA, Bianco-Miotto T, McAninch D, Goh Z, Anderson PH, et al. Reduced dietary calcium and vitamin D results in preterm birth and altered placental morphogenesis in mice during pregnancy. Reprod Sci. 2020;27: 1330–1339. doi: 10.1007/s43032-019-00116-2 32046423

[80] Tanriover MD, Oz SG, Sozen T. Ten-year follow-up in pregnancy and lactation-associated osteoporosis: sequential therapy with strontium ranelate and ibandronate. Spine J. 2015;15: 1164–1165. doi: 10.1016/j.spinee.2014.10.022 25925624

[81] Sanghvi TG, Harvey PW, Wainwright E. Maternal iron-folic acid supplementation programs: evidence of impact and implementation. Food Nutr Bull. 2010;31: S100–S107. doi: 10.1177/15648265100312S202 20715594

[82] Bezanson K, Isenman P. Scaling up nutrition: a framework for action. Food Nutr Bull. 2010;31: 178–186. doi: 10.1177/156482651003100118 20461915

[83] Liu D, Li S, Lei F, Zhao Y, Cheng Y, Dang S, et al. Associations between maternal calcium intake from diet and supplements during pregnancy and the risk of preterm birth in a Chinese population. Eur J Clin Nutr. 2021;75: 141–150. doi: 10.1038/s41430-020-00701-8 32814854

[84] Sanz-Salvador L, Garcia-Perez MA, Tarin JJ, Cano A. Bone metabolic changes during pregnancy: a period of vulnerability to osteoporosis and fracture. Eur J Endocrinol. 2015;172: R53–R65. doi: 10.1530/EJE-14-0424 25209679

[85] Willemse JPMM, Meertens LJE, Scheepers HCJ, Achten NMJ, Eussen SJ, Dongen MCV, et al. Calcium intake from diet and supplement use during early pregnancy: the expect study I. Eur J Nutr. 2020;59: 167–174. doi: 10.1007/s00394-019-01896-8 30661104 PMC7000487

[86] Bartelt A, Beil FT, Muüller B, Koehne T, Yorgan TA, Heine M, et al. Hepatic lipase is expressed by osteoblasts and modulates bone remodeling in obesity. Bone. 2014;62: 90–98. doi: 10.1016/j.bone.2014.01.001 24440515

[87] Kovacs CS, Ralston SH. Presentation and management of osteoporosis presenting in association with pregnancy or lactation. Osteoporos Int. 2015;26: 2223–2241. doi: 10.1007/s00198-015-3149-3 25939309

[88] Nakamura Y, Kamimura M, Ikegami S, Mukaiyama K, Komatsu M, Uchiyama S, et al. A case series of pregnancy- and lactation-associated osteoporosis and a review of the literature. Ther Clin Risk Manag. 2015;11: 1361–1365. doi: 10.2147/TCRM.S87274 26379439 PMC4567231

[89] Cormick G, Betran AP, Harbron J, Seuc A, White C, Roberts JM, et al. The effect of calcium supplementation on body weight before and during pregnancy in women enrolled in the WHO calcium and preeclampsia trial. Food Nutr Bull. 2020;41: 332–342. doi: 10.1177/0379572120944671 33200626 PMC11951462

[90] Koo WW, Hockman EM, Dow M. Palm olein in the fat blend of infant formulas: effect on the intestinal absorption of calcium and fat, and bone mineralization. J Am Coll Nutr. 2006;25: 117–122. doi: 10.1080/07315724.2006.10719521 16582027

[91] Petit V, Sandoz L, Garcia-Rodenas CL. Importance of the regiospecific distribution of long-chain saturated fatty acids on gut comfort, fat and calcium absorption in infants. Prostaglandins Leukot Essent Fat Acids. 2017;121: 40–51. doi: 10.1016/j.plefa.2017.05.007 28651696

[92] Alvarez FJ, Stella VJ. The role of calcium ions and bile salts on the pancreatic lipase-catalyzed hydrolysis of triglyceride emulsions stabilized with lecithin. Pharm Res. 1989;6: 449–457. doi: 10.1023/a:1015956104500 2762220

[93] Zhang L, Lookene A, Wu G, Olivecrona G. Calcium triggers folding of lipoprotein lipase into active dimers. J Biol Chem. 2005;280: 42580–42591. doi: 10.1074/jbc.M507252200 16179346

