# Supplementary material for: Low serum lipase levels in mothers of children with stunted growth indicate the possibility of low calcium absorption during pregnancy: A cross-sectional study in North Sumatra, Indonesia
Source: PLoS One. 2024 Jun 6;19(6):e0298253. doi: 10.1371/journal.pone.0298253 (PMC11156305; doi:10.1371/journal.pone.0298253)
Supplement: S1 Table — (PDF) [file pone.0298253.s001.pdf]

Table 1. Maternal Sociodemographic Characteristics and Pregnancy History.

Variable	Mothers of children with normal growth	Mothers of children with stunted growth	*p*
Age			
Mean ± SD (years)	32.41 ± 5.99	28.6 ± 6.25	0.05[a]
Age category (years):			
18–20	1 (3.1%)	1 (5.6%)	0.34[c]
20–30	12 (37.5%)	11 (61.1%)	
30–40	18 (56.3%)	6 (33.3%)	
40–50	1 (3.1%)	0	
Mother's occupation:			
Midwife	1 (3.1%)	0	0.46[c]
Teacher	1 (3.1%)	1 (5.6%)	
Housewife	24 (75.0%)	17 (94.4)	
Labour	1 (3.1%)	0	
Government employee	5 (15.6)	0	
Husband's occupation:			
Labour	2 (6.3%)	3 (16.7%)	0.56[c]
Teacher	2 (6.3%)	0	
Fisherman	10 (31.3%)	5 (27.8%)	
Small traders	17 (53.1%)	10 (55.6%)	
Government employee	1 (3.1%)	0	
Mother's education level:			
No education	0	1 (5.6%)	0.34[c]
Primary School	13 (40.6%)	8 (44.4%)	
Junior High School	4 (12.5%)	3 (16.7%)	
Senior High School	7 (21.9%)	5 (27.8%)	
Bachelor's degree	8 (25.0%)	1 (2.0%)	
Family income per month:			
Mean ± SD (USD)	93.19 ±62.17	92.73± 48.49	0.98[b]
Family income category:			
<RMW (USD 173.69)	27 (84.4%)	16 (88.9%)	1.00[c]
≥RMW (USD 173.69)	5 (15.6%)	2 (11.1%)	
Number of children:			
Mean ± SD (count)	2.56 ± 1.4	2.83 ± 1.5	0.90[b]
Age at first pregnancy:			
Mean ± SD (years)	24.38 ± 5.42	24.17 ± 7.08	0.18[b]
Problems during			

pregnancy:<br>Fever<br>Abdominal discomfort<br>Nausea and vomiting<br>Abdominal cramps<br>No problems	0<br>3 (9.4%)<br>2 (6.3%)<br>1 (3.1%)<br>26 (81.3%)	1 (5.6%)<br>0<br>3 (16.7%)<br>0<br>14 (77.8%)	0.26[c]
History of miscarriage:<br>Yes<br>No	 3 (9.4%)<br>29 (90.6%)	 1 (5.6%)<br>17 (94.4%)	1.0[c]
Antenatal care:<br>Mean ± SD (times per pregnancy)	6.84 ± 2.05	6.06 ± 1.89	0.19[a]

RMW: Regional minimum wage

[a] Independent t test

[b] Mann–Whitney U test

[c] Fisher's exact test

*significance: $p < 0.05$
