# Supplementary material for: Low serum lipase levels in mothers of children with stunted growth indicate the possibility of low calcium absorption during pregnancy: A cross-sectional study in North Sumatra, Indonesia
Source: PLoS One. 2024 Jun 6;19(6):e0298253. doi: 10.1371/journal.pone.0298253 (PMC11156305; doi:10.1371/journal.pone.0298253)
Supplement: S2 Table — (PDF) [file pone.0298253.s002.pdf]

Table 2. Maternal Anthropometric Data and Nutritional Intake.

Variable	Mothers of children with normal growth	Mothers of children with stunted growth	*p*
Mothers			
Body weight	56.94 ± 11.85	57.06 ± 10.29	0.84[b]
Height	155.41 ± 6.06	153.44 ± 4.60	0.21[a]
Body mass index	32.47 ± 4.16	24.14 ± 3.93	0.58[a]
Mother's body mass index category:			
Underweight	3 (9.4%)	1 (5.6%)	0.89[b]
Normal	15 (46.9%)	9 (50%)	
Overweight	6 (18.8%)	2 (11.1%)	
Obese 1	6 (18.8%)	5 (27.8%)	
Obese 2	2 (6.3%)	1 (5.6%)	
Mother's intake per day			
Calorie intake	1606.03 ± 298.8	1582.5 ± 269.5	0.78[a]
Carbohydrate intake	233.29 ± 47.7	220.34 ± 42.23	0.33[a]
Protein intake	61.28 ± 12.69	56.32 ± 14.59	0.24[a]
Fat intake	47.68 ± 18.74	55.61 ± 25.19	0.25[a]

[a] Independent t test

[b] Mann–Whitney U test

[c] Fisher's exact test

*significance: $p < 0.05$
