# Supplementary material for: Low serum lipase levels in mothers of children with stunted growth indicate the possibility of low calcium absorption during pregnancy: A cross-sectional study in North Sumatra, Indonesia
Source: PLoS One. 2024 Jun 6;19(6):e0298253. doi: 10.1371/journal.pone.0298253 (PMC11156305; doi:10.1371/journal.pone.0298253)
Supplement: S3 Table — (PDF) [file pone.0298253.s003.pdf]

Table 3. Anthropometric Data and Nutritional Intake of the Children.

Variable	Children with normal growth	Children with stunted growth	p
Age (months)	28.75 ± 18.4	31.89 ± 21.7	0.23[a]
Birth weight (g)	3200 ± 660	3100 ± 660	0.59
Exclusive breastfeeding			
Yes	19 (59.4%)	9 (50%)	0.56[c]
No	13 (40.6%)	9 (50%)	
Children growth Category			
Weight for age:			
Severe underweight	2 (6.3%)	2 (11.1%)	0.43[c]
Underweight	1 (3.1%)	2 (11.1%)	
Normal	27 (84.4%)	14 (77.8%)	
Obese	2 (6.3%)	0	
Weight for height			
Severe wasting	4 (12.5%)	0	0.11[c]
Wasting	2 (6.3%)	1 (5.6%)	
Normal	24 (75%)	12 (66.7%)	
Obese	2 (6.3%)	5 (27.8%)	
Child's intake per day			
Calorie intake	907.12 ± 351.81	791.29 ± 215.82	0.16[b]
Carbohydrate intake	207.38 ± 512.05	101.41 ± 38.19	0.25[b]
Protein intake	34.11 ± 15.19	32.38 ± 8.85	0.61[a]
Fat intake	31.37 ± 14.99	28.93 ± 10.77	0.51[a]

[a] Independent t test

[b] Mann–Whitney U test

[c] Fisher's exact test

*significance: $p < 0.05$
