# Supplementary material for: Low serum lipase levels in mothers of children with stunted growth indicate the possibility of low calcium absorption during pregnancy: A cross-sectional study in North Sumatra, Indonesia
Source: PLoS One. 2024 Jun 6;19(6):e0298253. doi: 10.1371/journal.pone.0298253 (PMC11156305; doi:10.1371/journal.pone.0298253)
Supplement: S4 Table — (PDF) [file pone.0298253.s004.pdf]

Table 4. Differences in the Vitamin, Mineral, and Enzyme Parameters of the Mothers.

Variable	Mothers of children with normal growth	Mothers of children with stunted growth	*p*
Calcium serum level (mg/dL)	8.82 ± 0.35	9.05 ± 0.37	0.03*[b]
Calcium category:			
Deficiency	2 (6.3%)	0(0%)	0.53[c]
Normal	30 (93.8%)	18 (100%)	
Serum iron level (mcg/dL)	90.25 ± 44.38	94.72 ± 28.91	0.67[a]
Iron category:			
Low	1 (3.1%)	0(0%)	0.54[c]
Normal	27 (84.4%)	17 (94.4%)	
High	4 (12.5%)	1 (5.6%)	
Serum zinc level (mcg/dL)	71.84 ± 7.97	75.0 ± 9.37	0.24[a]
Zinc category:			
Low	2 (6.3%)	1 (5.6%)	1.0[c]
Normal	30 (93.8%)	17 (94.4%)	
Serum vitamin D level (ng/mL)	19.0 ± 4.51	20.1 ± 5.14	0.45[a]
Vitamin D category:			
Deficiency	0 (0%)	1 (5.6%)	0.36[c]
Insufficiency	32 (100%)	17 (94.4%)	
Sufficiency	0 (0%)	0 (0%)	
Amylase pancreatic (U/L)	26.03 ± 5.11	25.44 ± 8.03	0.78[a]
Amylase category:			
Low	0 (0%)	0 (0%)	-
Normal	32 (100%)	18 (100%)	
Lipase (U/L)	20.06 ± 6.61	15.72 ± 6.01	0.03*[a]
Lipase category:			
Low	4 (12.5%)	7 (38.9%)	0.04*[c]
Normal	28 (87.5%)	11 (61.1%)	

[a] Independent t test

[b] Mann–Whitney U test

[c] Fisher's exact test

*significance: $p < 0.05$
