# Supplementary material for: Low serum lipase levels in mothers of children with stunted growth indicate the possibility of low calcium absorption during pregnancy: A cross-sectional study in North Sumatra, Indonesia
Source: PLoS One. 2024 Jun 6;19(6):e0298253. doi: 10.1371/journal.pone.0298253 (PMC11156305; doi:10.1371/journal.pone.0298253)
Supplement: S2 Data — (PDF) [file pone.0298253.s007.pdf]

PT. SARASWANTI INDO GENETECH

ONE STOP LABORATORY SERVICES

Main Office and Laboratory: Graha SIG Jl Rasamala No.20 Taman Yasmin Bogor 16113 INDONESIA
Jakarta Branch: Jl. Percetakan Negara No. 52 B RT 006/ RW 001 Kel. Rawasari, Kec. Cempaka Putih, Jakarta INDONESIA
Phone: (Bogor) +62-251-7532348 (**Jakarta**) +62-21-21479292 (**Surabaya**) 031-8678555 (**Semarang**) +62-81391706805 (**Hunting**) +62-82111516516 **Fax:** +62-251-7540927 – 7540928
www.siglaboratory.com

Bogor, November 11, 2020

No : SIG.CL.XI.2020.038840
Subject : Result of Analysis

To :
Dr. dr. Dina Keumala Sari
Jl. Setia Budi. Komp TASBIH 1, Blok G No. 29, Medan

Dear Sir/Madam,
As your order no : SIG.Mark.P.X.2020.001903 , herewith we send the result of analysis.

Thank you for your cooperation

Yours Faithfully,
PT. Saraswanti Indo Genetech

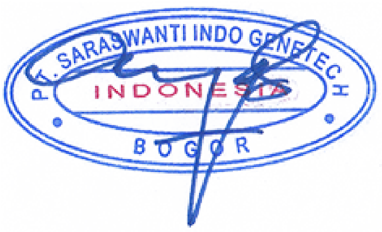

Robertus B.Aryo
Marketing Manager

PT. SARASWANTI INDO GENETECH
ONE STOP LABORATORY SERVICES

Main Office and Laboratory: Graha SIG Jl Rasamala No.20 Taman Yasmin Bogor 16113 INDONESIA
Jakarta Branch: Jl. Percetakan Negara No. 52 B RT 006/ RW 001 Kel. Rawasari, Kec. Cempaka Putih, Jakarta INDONESIA
Phone: (Bogor) +62-251-7532348 (**Jakarta**) +62-21-21479292 (**Surabaya**) 031-8678555 (**Semarang**) +62-81391706805 (**Hunting**) +62-82111516516 **Fax:** +62-251-7540927 – 7540928
www.siglaboratory.com

No. 28.1/F-PP/SMM-SIG
Revisi : 4

RESULT OF ANALYSIS

Laporan Hasil Pengujian
SIG.LHP.XI.2020.119458

**I. Number / Nomor**	
1.1. Order No. / No. Order	: SIG.Mark.P.X.2020.001903
**II. Principal / Pelanggan**	
2.1. Name / Nama	: Dr. dr. Dina Keumala Sari
2.2. Address / Alamat	: Jl. Setia Budi. Komp TASBIH 1, Blok G No. 29, Medan
2.3. Phone / Telepon	: 081397177693
2.4. Contact Person / Personil Penghubung	: Dr. dr. Dina Keumala Sari, MG, SpGK
**III. Sample / Contoh Uji**	
3.1. Sample Code / Kode Sampel	: -
3.2. Batch Number / No Batch	: -
3.3. Lot Number / No Lot	: -
3.4. Packaging / Kemasan	: -
3.5. Production Date / Tanggal Produksi	: -
3.6. Expire Date / Tanggal Kadaluarsa	: -
3.7. Factory Name / Nama Pabrik	: -
3.8. Factory Address / Alamat Pabrik	: -
3.9. Trade Mark / Nama Dagang	: -
3.10. Sample Name / Nama Sample	: Tanah
3.11. Other Information / Keterangan Lain	: -
3.12. Date of Sampling / Tanggal Sampling	: -
3.13. Date of Received / Diterima	: October 30, 2020
3.14. Date of Analysis / Tanggal Uji	: November 02, 2020 - November 10, 2020
3.15. Type of Analysis / Jenis Uji	: Enclosed
**IV. Result / Hasil Uji**	

Result of analysis on page I

The results of these tests relate only to the sample(s) submitted. This report shall not be reproduced except in full context, without the written approval of PT. Saraswanti Indo Genetech

PT. SARASWANTI INDO GENETECH

ONE STOP LABORATORY SERVICES

Main Office and Laboratory: Graha SIG Jl Rasamala No.20 Taman Yasmin Bogor 16113 INDONESIA
Jakarta Branch: Jl. Percetakan Negara No. 52 B RT 006/ RW 001 Kel. Rawasari, Kec. Cempaka Putih, Jakarta INDONESIA
Phone: (Bogor) +62-251-7532348 (**Jakarta**) +62-21-21479292 (**Surabaya**) 031-8678555 (**Semarang**) +62-81391706805 (**Hunting**) +62-82111516516 **Fax:** +62-251-7540927 – 7540928
www.siglaboratory.com

No. 28.1/F-PP/SMM-SIG
Revisi : 4

Result of Analysis
No : SIG.LHP.XI.2020.119458

No.	Parameter	Unit	Result	Limit Of Detection	Method
1	Magnesium	mg / 100 g	87.20	-	18-13-1/MU/SMM-SIG (ICP OES)
2	Potassium	mg / 100 g	66.96	-	18-13-1/MU/SMM-SIG (ICP OES)
3	Phosphorus	mg / kg	251.92	-	18-13-1/MU/SMM-SIG (ICP OES)
4	Nitrogen	%	0.10	-	18-8-31/MU/SMM - SIG (Kjeltec)

Bogor, November 11, 2020
PT. Saraswanti Indo Genetech

Dwi Yulianto Laksono, S.Si
Laboratory Manager

Result of analysis on page II

The results of these tests relate only to the sample(s) submitted. This report shall not be reproduced except in full context, without the written approval of PT. Saraswanti Indo Genetech
