# Supplementary material for: Low serum lipase levels in mothers of children with stunted growth indicate the possibility of low calcium absorption during pregnancy: A cross-sectional study in North Sumatra, Indonesia
Source: PLoS One. 2024 Jun 6;19(6):e0298253. doi: 10.1371/journal.pone.0298253 (PMC11156305; doi:10.1371/journal.pone.0298253)
Supplement: S1 Checklist — (PDF) [file pone.0298253.s008.pdf]

STROBE Statement—Checklist of items that should be included in reports of ***cross-sectional studies***

	Item No	Recommendation	Page/Line
**Title and abstract**	1	(*a*) Indicate the study's design with a commonly used term in the title or the abstract	1-2/1-3 (title) and 128-129 (abstract)
		(*b*) Provide in the abstract an informative and balanced summary of what was done and what was found	2/124-141
**Introduction**			
Background/rationale	2	Explain the scientific background and rationale for the investigation being reported	3-5/242-511
Objectives	3	State specific objectives, including any prespecified hypotheses	5/504-508
**Methods**			
Study design	4	Present key elements of study design early in the paper	5/515-521
Setting	5	Describe the setting, locations, and relevant dates, including periods of recruitment, exposure, follow-up, and data collection	6/567-577
Participants	6	(*a*) Give the eligibility criteria, and the sources and methods of selection of participants	7/622-636
Variables	7	Clearly define all outcomes, exposures, predictors, potential confounders, and effect modifiers. Give diagnostic criteria, if applicable	5/504-508<br>5/516-521<br>7/626-627-confounding
Data sources/ measurement	8*	For each variable of interest, give sources of data and details of methods of assessment (measurement). Describe comparability of assessment methods if there is more than one group	8-12/655-1188
Bias	9	Describe any efforts to address potential sources of bias	12/1178-1180
Study size	10	Explain how the study size was arrived at	6/560-566
Quantitative variables	11	Explain how quantitative variables were handled in the analyses. If applicable, describe	8-13/655-1235

		which groupings were chosen and why	
Statistical methods	12	(*a*) Describe all statistical methods, including those used to control for confounding	12-13/1190-1234
		(*b*) Describe any methods used to examine subgroups and interactions	13/1227-1234
		(*c*) Explain how missing data were addressed	7/633-636<br>This study completed all the data
		(*d*) If applicable, describe analytical methods taking account of sampling strategy	6/578-620
		(*e*) Describe any sensitivity analyses	All the equipment already through sensitivity analysis
**Results**			
Participants	13*	(a) Report numbers of individuals at each stage of study—eg numbers potentially eligible, examined for eligibility, confirmed eligible, included in the study, completing follow-up, and analysed	7/622-636
		(b) Give reasons for non-participation at each stage	Not applicable
		(c) Consider use of a flow diagram	7/622-636, only narrative explanation
Descriptive data	14*	(a) Give characteristics of study participants (eg demographic, clinical, social) and information on exposures and potential confounders	6/571-577
		(b) Indicate number of participants with missing data for each variable of interest	No missing data
Outcome data	15*	Report numbers of outcome events or summary measures	13-20/1244-1574
Main results	16	(*a*) Give unadjusted estimates and, if applicable, confounder-adjusted estimates and their precision (eg, 95% confidence interval). Make clear which confounders were adjusted for and why they were included	Already explain about confounders
		(*b*) Report category boundaries when continuous variables were	13-20/1244-1574

		categorized	
		(*c*) If relevant, consider translating estimates of relative risk into absolute risk for a meaningful time period	13-20/1244-1574
Other analyses	17	Report other analyses done—eg analyses of subgroups and interactions, and sensitivity analyses	13-20/1244-1574
**Discussion**			
Key results	18	Summarise key results with reference to study objectives	21-26/ 1621-2225
Limitations	19	Discuss limitations of the study, taking into account sources of potential bias or imprecision. Discuss both direction and magnitude of any potential bias	13-20/1244-1574
Interpretation	20	Give a cautious overall interpretation of results considering objectives, limitations, multiplicity of analyses, results from similar studies, and other relevant evidence	13-20/1244-1574
Generalisability	21	Discuss the generalisability (external validity) of the study results	13-20/1244-1574
**Other information**			
Funding	22	Give the source of funding and the role of the funders for the present study and, if applicable, for the original study on which the present article is based	In cover letter. PlosOne editor ask to move the funding information to cover letter: This work was funded by Ministry of Education and Culture Republic of Indonesia under the World Class University Program of Universitas Sumatera Utara Year 2020. The Grant Number 1879/UN5.1.R/SK/PPM/2020 and the project was run as my research project.

*Give information separately for exposed and unexposed groups.

Note: An Explanation and Elaboration article discusses each checklist item and gives methodological background and published examples of transparent reporting. The STROBE checklist is best used in conjunction with this article (freely available on the Web sites of PLoS Medicine at http://www.plosmedicine.org/, Annals of Internal Medicine at http://www.annals.org/, and Epidemiology at http://www.epidem.com/). Information on the STROBE Initiative is available at www.strobe-statement.org.
