# Supplementary material for: Low serum lipase levels in mothers of children with stunted growth indicate the possibility of low calcium absorption during pregnancy: A cross-sectional study in North Sumatra, Indonesia
Source: PLoS One. 2024 Jun 6;19(6):e0298253. doi: 10.1371/journal.pone.0298253 (PMC11156305; doi:10.1371/journal.pone.0298253)
Supplement: Attachment — Submitted filename: Response to Reviewers.pdf [file pone.0298253.s011.pdf]

EVALUATING THE EFFECT OF LOW LIPASE ENZYME SERUM ON 5 STUNTING PROBLEMS IN NORTH SUMATRA, INDONESIA

-Consider revising the title. I think it does not capture the entirety of the whole article. The use of effect may not be suitable since the study is cross-sectional.
<u>Response:</u>
The title already revised with the title that better reflection to the results.
The title also add: A cross sectional study to let the other researcher can capture the entirety of the whole article by seing the title.
Please check in the line 1-3

-The journal article needs language editing.
<u>Response:</u>
Yes, I already upload the English editing summary, with American Journal Editing service

-The introduction did not provide substantive reasons of why mother's serum lipase enzyme is the focus of the article.
<u>Response:</u>
Yes, I already add statement about this substantive reasons. Based on previous research there is lack of studies about enzyme that related to nutrition absorption, and in this research we focus on two enzymes which are amylase and lipase. We also interest with lipase because of lipase affect fat absorption that will also affect calcium absorption.
Please check in the line 356-358 and follow on the line 358-362.

Methodology

-Did you collect low birth weight data? I think this variable is important in your research.
<u>Response:</u>
Yes, We collect these data. We presented in the table 3, row 2. We also add analysis in the line 403-405. Probably because of the stunting children only 18 children, compare with normal children 32, so this could results no significance difference. This is from our view about this study, the probable answer to this non-significance results.

-page 8, line 200-205 – Consider adding the validation procedures.
<u>Response:</u>
Yes, we already add this validation information. NutriSurvey application already.
Please check on the line 184-188.

- Are your subjects randomly selected? If not, can your data be analyzed using standard statistical test?
<u>Response:</u>
Yes it was randomly selected. I already add explanation about sample selected process. Please check in the line 628-632.

Results

-Table 1 – Add information on US$ for income
Response:
Yes please check in the table 1 page 14-15, we already revise it

-Table 2 – Consider using BMI instead of Weight for height for mother's nutritional status.
Response:
Yes we already revised it, please check in table 2, page 16-17, we already revise it

-Table 2 – Consider separating the data for mother and children.
Response:
Yes, we already revised it in table 2 and 3, already separate, page 16-17 (table 2) and 17-18 (table 3).

-Table 3 – Consider separating the results of serum mineral level from mineral status by a secondary line.
Response:
Yes, we already revise it in table 4. In the page 19. Secondary line and we put space to make the readers of this article easier to understand the table.

Discussion

-Did you use previous articles on post-natal effect on stunting when you drafted the proposal for this research?
Response:
Most of the previous article focusing in pre-natal effect on stunting, we lack of studies on post-natal effect, but, we still focusing in mother's nutrition status post-natal effect. The nutritional status of the mother is important, not only pre-natally including observing the mother's body mass index or height, but also the vitamin-mineral and enzyme status of the productive mother after giving birth. This can describe the condition of the mother pre-natally and during pregnancy.

However, this is a deeper concern because we should pay attention to the status of vitamins, minerals and enzymes because the possibility of a productive mother becoming pregnant again is very large. Moreover, mothers think that they do not pay attention to the birth distance between one child and another. This condition is one of the reasons why examination of vitamin-mineral and enzyme status is the main focus and is also carried out after birth.

Is this research output a part of a bigger research?
Response:
No, this is an independent study with the hypothesis that to find the difference of vitamin-mineral and enzyme between stunting and normal group in post natal mother. After this publication, we will continue to find the exact mechanism and the different methodology to find this relationships

or effect lipase on stunting. This is a base data to expand the next research. If we can find more results, this will help to understand stunting problem and reduce stunting rates in Indonesia. We will move to the next steps, with another aims and hypothesis.

-The age group of children is from 2-60 months. Did you analyze the data between mothers of less than 2 years old (breastfeeding) and those 2 years old and above. In addition, breastfeeding mothers may have different mechanism than non-breastfeeding mothers.

Response:

Yes we will analyze it, but we still consider for sample size, we need bigger sample size to have normal distribution data and we can find the mechanism for non-breastfeed mother, but for this study, still we lack of information about this breastfeeding mechanism. We do hope for the next research after this publication.

-What are the confounders in this research?

Response:

Confounding factors in this study are mothers with gastrointestinal disorders and mothers with metabolic disorders, we control it in inclusion and exclusion criterias. Please see the line 626-627.

-There were many 'probably' in the article. Consider expounding the biological mechanism of lipase given that calcium is higher among mother of non-stunted children.

Response:

Yes, we already explain the mechanism, we hope it will open other research about this. Please check in line 2065-2075.

- Lines 354-378 the mechanism explained is for pregnant mothers (which is not the subject of your study). Can you find a more relevant mechanism for post-natal women?

Response:

Yes, we discuss about this that it is very important to have normal status nutrition for every productive mother, along every stage of her productive period, not only pre-natally including observing the mother's body mass index or height, but also the vitamin-mineral and enzyme status of the productive mother after giving birth. This can describe the condition of the mother pre-natally and during pregnancy. However, this is a deeper concern because we should pay attention to the status of vitamins, minerals and enzymes because the possibility of a productive mother becoming pregnant again is very large. Moreover, mothers think that they do not pay attention to the birth distance between one child and another. This condition is one of the reasons why examination of vitamin-mineral and enzyme status is the main focus and is also carried out after birth.
We already put this analysis in page 2076-2196.

Line 386-387 - "We also did not you did not test mothers when they were carrying babies, so it might their lipase levels have changed in the period intervening birth and our study. Another limitation is that there were no data on the maternal parathyroid hormone, calcium, vitamin

D, and lipase levels in children." Do you think your explanations and conclusion are valid given these limitations?

Response:

In this study we present the results of the research using the correct methodology, it is hoped that we can provide valid results. as long as these results pave the way for further research, it is hoped that new findings will be obtained to overcome stunting, especially in Indonesia. The prevalence of stunting has decreased in accordance with government programs, but it still exists and is growing. It is hoped that this research can open up other research to examine the causes. Over the last 10 years, improvements in nutritional status have been implemented, but it is possible that there are other causes that can be explored. Something new and understandable if we get results that need to be developed.
