# Supplementary material for: Low serum lipase levels in mothers of children with stunted growth indicate the possibility of low calcium absorption during pregnancy: A cross-sectional study in North Sumatra, Indonesia
Source: PLoS One. 2024 Jun 6;19(6):e0298253. doi: 10.1371/journal.pone.0298253 (PMC11156305; doi:10.1371/journal.pone.0298253)
Supplement: Attachment — Submitted filename: Response_to Reviewers .docx [file pone.0298253.s009.docx]

**RESPONSE TO REVIEWERS**

Comments	:The reviewers have raised a number of minor concerns. They feel that the title should be modified to better reflect the results, and the discussion should be more concise. The reviewers also note some typographical errors that need attention.

Revised	:Yes, I already changed the title: Low Levels of Lipase Serum In Mothers Who Have Stunting Children Provide The Possibility of Low Calcium Absorption During Pregnancy in North Sumatra, Indonesia (in line 4-6).

Comment

Revisions being requested Title: Cross-sectional study design does not evaluate the “effect” of something because data on both exposure and outcome are collected at the same time and there is no way to determine if exposure led to an outcome.
The word “problems” is not relevant in the title.

Revised	:Yes, I already revised it to title that showed the results of the study

**Revision:**

**Abstract**

1. Lines 33 - 35: Grammatical error, revise the sentence “The objectives …. “.

Revised:

This study aimed to investigate the differences in sociodemographic factors, mineral, vitamin, and enzymes parameters associated with the occurrence of stunting in mothers with normal and stunting children. (line 34-36)

**Introduction**

2. Lines 78 - 79: The statement “To the best … “ is misleading. How does this statement agree with lines 69 - 70? Do you mean a study like yours was not conducted in the study area before?

Revised:

I already change it with this statement: “Based on previous research, there is still more needed to explore the role of vitamins, minerals, and enzymes to prevent stunting” (line 79-80)

I do agree, I hope it will not mislead the goals of this study and the reader will not misinterpretation about this study.

3. Materials and Methods
   Lines 94 and 123: The sections “study design and participants” and “participants” should be reorganised.

Revised:

I already revised it, to reorganized the section, please check in line 95 and 118.

4. Lines 116 – 121: Please move these words under the sub-section “Ethical considerations” which should be the last sub-section of “Materials and methods”.

Revised:

I already revised it, please check in line 215.

5. Lines 136 - 137: You stated that soil and water content were checked in a laboratory but I did not see any results on these.

Revised:

Yes, I already add the information about the soil and water, please check in line 133-143

6. Lines 140 – 147: There is no need to capitalize “microtoise”.

Revised:

Yes, I already revised it, please check 146-156.

7. Lines 166 – 169: Make reference to anthropometric z-scores in the definition of wasting, stunting and underweight.

Revised:

Yes, I already revised it, please check in line 172-179.

8. Line 214: Insert “and” between error and 95% confidence interval.

Revised:

Yes, I already revised it, please check in line 224

9. Table 1: Add unit of measurement to category of age.

Revised:

Yes, I already revised it in table 1

10. Table 1: Last education, Define SD, SMP, SMA and S1.

Revised:

Yes, I already revised it in table 1

11. Table 1: Family income per month, give US dollar equivalent of amount beneath the table.

Revised:

Yes, I already revised them. I also revise in line 252.

12. Lines 242 - 243: Grammatical error, revise sentence “The group …”.

Revised:

Yes, I already revised in line 261

13. Table 2: Children growth category, I do not see rationale for including height-for-age under this label as the children were divided into two groups using height-for-age z-score.

Revised:

Yes I already revised it in the table 2.

**Discussion**

14. Line 331: Remove the “of” at the end of the line

Revise:

Yes, I already revised it in line 347-348. I already delete ‘of”.

15. Line 377: Mechanism is incorrectly spelt.

Revise:

Yes, I already revised it in line 393

16. Lines 385 - 243: Grammatical error, revise sentence “We also …”.

Revise:

Yes, please check in line 359: “Mothers with normal growth children” to “mothers with normal children group”

Yes please check in line 401: “We also did not you did not test mothers” to “we did not test the mothers.

17. Lines 390 - 392: The word “need” is not necessary in that sentence.

Revise:

Yes, I already revised it. Line 406

Reviewer #2: The manuscript is well written in clear English, however some typographical errors that need attention are highlighted below:

Line 113: …”height per age”… this could read better as “ …height for age…”

Revised: yes, thank you, I already revised it to all the manuscript.

Line 119: How did the authors decide on who to use verbal consent and who to use written consent? What rationale was used to decide?

Comments: we used for **written and verbal informed consent in this study to all the subject. We did not separate or choose which subject use the verbal and written. We explained all the protocol to the subject, and we ask for agreement from the subject verbally but we also asked subject to read and signed the approval after explanation letter.**

Line 128: What could have been the result of recruiting clients for the study as described? Did the authors consider the risk of selection bias? How was this minimized or mitigated?

Comments:

Yes, in this study we selected subjects according to the inclusion criteria, namely mothers of a certain age who have stunted children and mothers who have normal children. This will introduce bias, but we will encounter difficulties if we use a random sampling system. this is because the number of participation of mothers with stunting children is different from that of mothers in the normal group. This selection bias cannot be controlled, it can only be prevented, but we did try to improve the quality of examinations and interviews properly and correctly so that the results of this study can be accounted for and the results of the sample can describe the results in a population.

Line 129: Was enrolment done before applying the exclusion criteria?

Comments:

Yes, it is true that registration is done before applying the exclusion criteria, inclusion criteria are carried out first and then exclusion criteria are applied. Therefore, after passing the exclusion criteria, there was a decrease in the number of samples. a total of twelve samples were excluded for several reasons.

Line 130-131: What was the rationale for dividing the subjects into the two groups?

Comments:

the reason to dividint the subjects into the two groups is to see the strength of the causal relationship. It is hoped that with good planning, careful implementation, and proper analysis, this study can make a meaningful contribution.

Line 242: …were taller be higher than… : correct the typos

Revised:

Yes, I already revised it please check in line 261.

Line 263: try and keep discussion out of the results section.

Revised:

I already revised it by deleting the sentences that should in discussion. In line 281-283.

“This result was interesting because there was a significant difference in serum calcium levels in the group of mothers who have normal and stunted children, but it appeared that low serum calcium levels were in the group of mothers with normal children (*p* = 0.03*,* mean difference±standard error (SE)= 0.23±0.12, 95% CI: 0.02–0.45)”.

Table 3: For vitamin D serum level, how did the authors define “Deficiency” and “Insufficiency” in this study?

Comments:

Yes, thank you. In line 207-209 in Methods section, in laboratory measurement, there were already state that: “The results of the examination of vitamin D levels were considered deficient in the range of serum 25 (OH) D levels, namely <10 ng/mL, while the insufficient category was 10–29 ng/ml, and the sufficient category was 30–100 ng/mL”.

Line 291: …..been completely work in this area….: needs correction.

Revised:

Yes I already revised it in line 313-314.

Line 377: “mechanisms” should have no “s” and instead of understand could authors could use another word for clarity.

Comments:Yes, I already revise it, it is also mean for this explanation can make the reader to understand about the role of lipase.

Line 385-386: repetition of words should be corrected and revise sentence for clarity.

Revised:

I already revised it, please check in line: 408.

**Results:**

The presentation of results does not clearly bring out what is stated in the title: “Evaluating the effect of low lipase enzyme serum on stunting problems in North Sumatra, Indonesia”. There seems to be some disconnect between the two.

Comments:

Yes I already revised the tittle with:

Low Levels of Lipase Serum In Mothers Who Have Stunting Children Provide The Possibility of Low Calcium Absorption During Pregnancy in North Sumatra, Indonesia

**Discussion:**

The discussion is rather long which leads to a tendency to include a lot of information.

Comments:

Yes, already revised it, we already deleted in paragraph 2 to reduce the discussions.
