# Supplementary material for: Low serum lipase levels in mothers of children with stunted growth indicate the possibility of low calcium absorption during pregnancy: A cross-sectional study in North Sumatra, Indonesia
Source: PLoS One. 2024 Jun 6;19(6):e0298253. doi: 10.1371/journal.pone.0298253 (PMC11156305; doi:10.1371/journal.pone.0298253)
Supplement: Attachment — Submitted filename: Comments_LSA.docx [file pone.0298253.s010.docx]

EVALUATING THE EFFECT OF LOW LIPASE ENZYME SERUM ON 5 STUNTING PROBLEMS IN NORTH SUMATRA, INDONESIA

-Consider revising the title. I think it does not capture the entirety of the whole article. The use of effect may not be suitable since the study is cross-sectional.

-The journal article needs language editing.

-The introduction did not provide substantive reasons of why mother’s serum lipase enzyme is the focus of the article.

Methodology

-Did you collect low birth weight data? I think this variable is important in your research.

-page 8, line 200-205 – Consider adding the validation procedures.

- Are your subjects randomly selected? If not, can your data be analyzed using standard statistical test?

Results

-Table 1 – Add information on US$ for income

-Table 2 – Consider using BMI instead of Weight for height for mother’s nutritional status.

-Table 2 – Consider separating the data for mother and children.

-Table 3 – Consider separating the results of serum mineral level from mineral status by a secondary line.

Discussion

-Did you use previous articles on post-natal effect on stunting when you drafted the proposal for this research? Is this research output a part of a bigger research?

-The age group of children is from 2-60 months. Did you analyze the data between mothers of less than 2 years old (breastfeeding) and those 2 years old and above. In addition, breastfeeding mothers may have different mechanism than non-breastfeeding mothers.

-What are the confounders in this research?

-There were many ‘probably’ in the article. Consider expounding the biological mechanism of lipase given that calcium is higher among mother of non-stunted children.

- Lines 354-378 the mechanism explained is for pregnant mothers (which is not the subject of your study). Can you find a more relevant mechanism for post-natal women?

Line 386-387 - “We also did not you did not test mothers when they were carrying babies, so it might their lipase levels have changed in the period intervening birth and our study. Another limitation is that there were no data on the maternal parathyroid hormone, calcium, vitamin D, and lipase levels in children.” Do you think your explanations and conclusion are valid given these limitations?
